# 2014. Central Line - Associated Bloodstream Infections and Their Increase With the COVID-19 Pandemic at Veterans Affairs in San Antonio, Texas

**DOI:** 10.1093/ofid/ofac492.1638

**Published:** 2022-12-15

**Authors:** Divya Chandramohan, Delvina Ford, Erica Beck, Lauren N Garza, Kelly R Reveles, Jose Cadena

**Affiliations:** UT Health-San Antonio, San Antonio, Texas; South Texas Veterans Health Care System, San Antonio, Texas; South Texas Veterans Health Care System, San Antonio, Texas; South Texas Veteran Health Care System, San Antonio, Texas; The University of Texas at Austin, San Antonio, Texas; South Texas Veterans Health Care System/ UT Health San Antonio, San Antonio, Texas

## Abstract

**Background:**

Coronavirus disease-19 (COVID-19) has been associated with an increase in healthcare-associated infections (HAI). This increase is likely multifactorial (i.e. higher hospitalization rates, COVID-19 and post-COVID-19 complications, lower staffing, delayed care among others). The objective of this study was to determine the association between COVID-19 hospitalization rates and central line- associated blood stream infections (CLABSI).

**Methods:**

We conducted a retrospective study in acute care unit hospitalizations in a Veterans Affairs (VA) hospital in San Antonio, Texas from October 2017 to December 2021. Individuals over 18 years of age admitted with a new diagnosis of COVID-19, determined by the severe acute respiratory syndrome coronavirus-2 (SARS-CoV-2) polymerase chain reaction (PCR) were included in the study. CLABSIs were defined by the National Healthcare Safety Network (NHSN) criteria for laboratory confirmed bloodstream infections. Pearson correlation was used to determine correlation of CLABSI and COVID-19 disease hospitalization rates. CLABSI rates were also compared pre-COVID-19 (Oct 2017-Feb 2020) to COVID-19 (Mar 2020-Dec 2021) periods using the chi-square test.

**Results:**

During the study period, a total of 0.69 CLABSIs per 1,000 central line days occurred in the pre-COVID-19 period compared to 1.98 per 1,000 in the COVID-19 period (p=0.004). There was a significant correlation between CLABSI and ICU COVID-19 hospitalization rates (R=0.459; p=0.001) as well as CLABSI and acute care COVID-19 hospitalization rates (R=0.341; p=0.014). During the COVID-19 period only, there continued to be a significant correlation between CLABSI and COVID-19 ICU hospitalization rates (R=0.426; p=0.048).

**Conclusion:**

CLABSI rates significantly increased during the COVID-19 period compared to the pre-COVID-19 period and CLABSI rates were significantly correlated with COVID-19 ICU and acute care hospitalizations. Accounting for this variable allows us to factor in impact of post-COVID-19 related complications and association with CLABSI rate. We urge for careful implementation of HAI prevention strategies during the pandemic. Awareness of anticipated increase is important in allocating resources essential for prevention of HAIs.

**Disclosures:**

**All Authors**: No reported disclosures.

